# Variance Component Analysis of a Multi-Site Study for the Reproducibility of Multiple Reaction Monitoring Measurements of Peptides in Human Plasma

**DOI:** 10.1371/journal.pone.0014590

**Published:** 2011-01-26

**Authors:** Jessie Q. Xia, Nell Sedransk, Xingdong Feng

**Affiliations:** 1 National Institute of Statistical Sciences, Research Triangle Park, North Carolina, United States of America; 2 Department of Statistics, Duke University, Durham, North Carolina, United States of America; Cuban Neuroscience Center, Cuba

## Abstract

**Background:**

In the Addona *et al.* paper (Nature Biotechnology 2009), a large-scale multi-site study was performed to quantify Multiple Reaction Monitoring (MRM) measurements of proteins spiked in human plasma. The unlabeled signature peptides derived from the seven target proteins were measured at nine different concentration levels, and their isotopic counterparts were served as the internal standards.

**Methodology/Principal Findings:**

In this paper, the sources of variation are analyzed by decomposing the variance into parts attributable to specific experimental factors: *technical replicates*, *sites*, *peptides*, *transitions within each peptide*, and higher-order interaction terms based on carefully built mixed effects models. The factors of *peptides* and *transitions* are shown to be major contributors to the variance of the measurements considering heavy (isotopic) peptides alone. For the light (^12^C) peptides alone, in addition to these factors, the factor of study*peptide also contributes significantly to the variance of the measurements. Heterogeneous peptide component models as well as influence analysis identify the outlier peptides in the study, which are then excluded from the analysis. Using a log-log scale transformation and subtracting the heavy/isotopic peptide [internal standard] measurement from the peptide measurements (i.e., taking the logarithm of the peak area ratio in the original scale establishes that), the MRM measurements are overall consistent across laboratories following the same standard operating procedures, and the variance components related to *sites*, *transitions* and higher-order interaction terms involving *sites* have greatly reduced impact. Thus the heavy peptides have been effective in reducing apparent inter-site variability. In addition, the estimates of intercepts and slopes of the calibration curves are calculated for the sub-studies.

**Conclusions/Significance:**

The MRM measurements are overall consistent across laboratories following the same standard operating procedures, and heavy peptides can be used as an effective internal standard for reducing apparent inter-site variability. Mixed effects modeling is a valuable tool in mass spectrometry-based proteomics research.

## Introduction

Mass spectrometry-based proteomics has emerged as one of the fundamental experimental means in a broad range of application fields, including protein biomarker studies [1]–[2], environmental studies [3], etc. Yet it has also been extensively criticized as a technique with poor repeatability and reproducibility [4]. In a thoughtful paper on analysis of shotgun proteomics [5], Eckel-Passow *et al.* discuss inherent difficulties in achieving good repeatability and reproducibility even presuming technically superb mass spectrometry; and they explain the consequent challenges for statistical analysis of shotgun proteomics data. For Multiple Reaction Monitoring (MRM) measurements, the issues are somewhat different and provide the opportunity to utilize the logic of [statistical] variance components analysis to distinguish among sources of variability that can impair both reliability and reproducibility.

In order to address the issues of reliability, reproducibility, precision and instrument capability at multiple levels (within laboratory, between laboratory, instrument-to-instrument, sample-to-sample, limits of dectection (LoD) and quantitation (LoQ)), a large-scale multi-site study was conducted to quantify MRM measurements of a standarized mix of proteins in human plasma [6]. The data analysis reported by Addona *et al.* [6] was done at a micro level: linear regression models were fitted for individual peptide groups measured at each site. Coefficients of variation (CV) were then computed and reported for the linear response, percent recovery, limites of dectection (LoD) and quantitation (LoQ).

The present paper addresses these same issues for MRM, using the publically available data from the interlab study [6] as illustration. Specifically, a variance components analysis of both spiked-in control and test sample data partitions the variation in peptide measurements into components that are attributable to the several experimental factors including *sites*, *peptides*, *transitions* and *sub-studies*. The analysis of the control data (heavy/isotopic peptides) establishes the experiment's reliability and reproducibility by the consistency of results. The analysis of test samples then eliminates variation due to extraneous sources from the calculation of the calibration curve and the precision estimates for that curve.

Mixed effects models are powerful and flexible tools for this purpose [7]; so these are forumulated here as the basis for an in-depth analysis of the variance components in the study. The discussion of the rationale behind the specific choices of fixed vs. random effects is provided in the Methods section.

This paper may be read in two ways, depending on the interests of the reader. First, it may be read as a report of detailed results of the interlab experiments [6]. Second, it may be read as a case study or tutorial in the use of variance components analysis for MRM calibration experiments. In the Methods section, the organization of the statistical analysis is laid out stepwise to give an overview of the analysis process that is subsequently presented in detail.

## Methods

### 1) The study

Addona *et al.* [6] conducted their multi-lab experiment(8 sites) using the technology of Multiple Reaction Monitoring (MRM) coupled with stable isotope dilution mass spectrometry (SID-MS) for the measurement of seven target proteins (11 unlabeled signature peptides, i.e., 11 peptide groups) spiked into human plasma. All the sites used the same protein mixture and spike-ins, and followed the same standard operating procedure (SOP, refer to Supplementary Document in [6]). Seven of them used 4000 QTRAP mass spectrometers. The eighth site used a different type of mass spectrometer (ThermoFisher Quantum Ultra triple quadrupole), which was excluded from our analysis.

**Figure 1:**
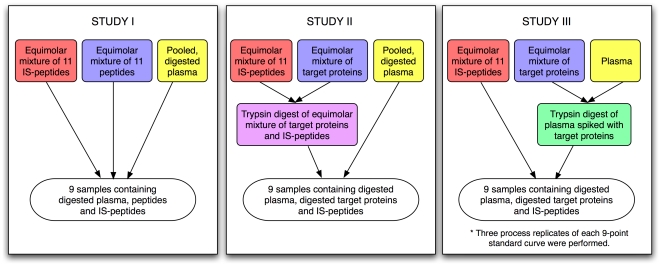
Illustration of three sub-studies in the study. Note that the complexity was increased from Study I to Study II to Study III.

The study comprised three sub-studies: Studies I, II and III, as shown in the overall study design diagram in Figure 1. Study I was conducted under the most controlled conditions, whereas Study III was closest to a real experiment where almost all sources of variations were involved. They were designed so that study complexity as well as experimental variations increased from Study I to Study III. In Study I, the light (^12^C) and heavy (isotopic) peptides were spiked into the digested human plasma. In Study II, the digested proteins were spiked into the digested human plasma. In Study III, the mixture of intact proteins and human plasma was digested followed by mixing with the isotopic peptide groups. Study III had three process/technical replicates. The light peptide groups were measured over 9 different concentration levels, whereas the heavy/isotopic peptide groups were at the same concentration throughout to serve as an internal standard. For each peptide group, three transitions were monitored; and for each transition, four replicates were acquired. Therefore, excluding the nested factor of transitions, each sub-study was a full factorial design [8] for the remaining factors: studies (3), sites (7), and peptides (11). What's shown in each pair of parenthesis is the number of levels for each factor.

### 2) Template for the Analysis

**Figure 2:**
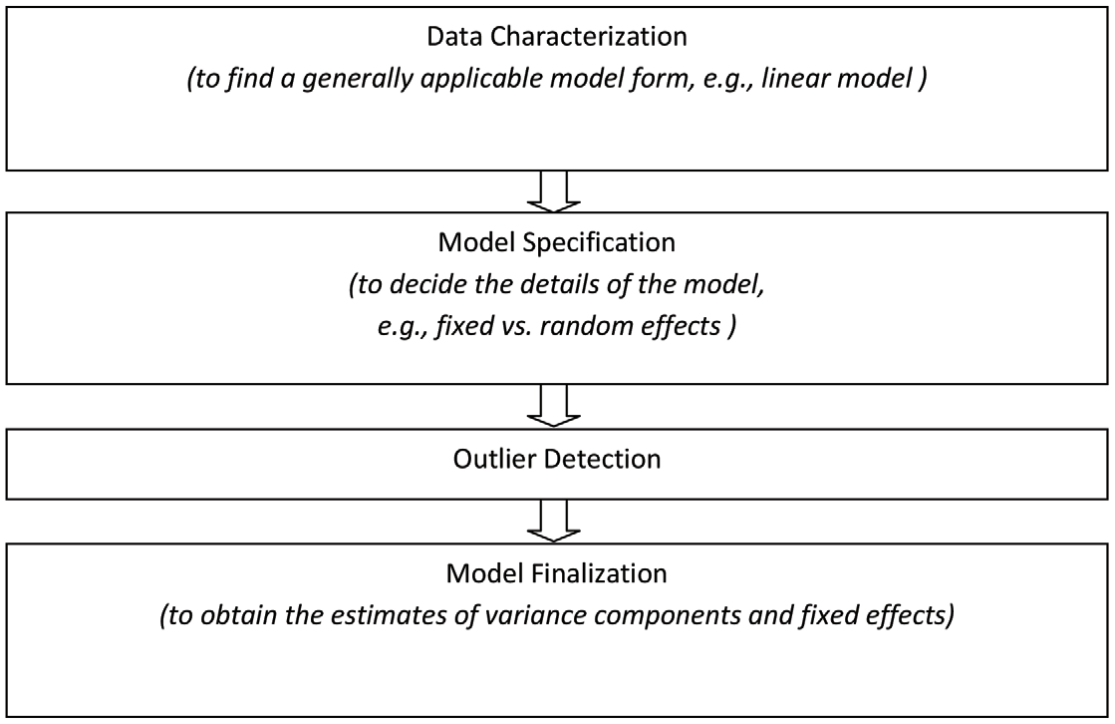
Sketch flowchart of variance component analysis procedure.

Figure 2 illustrates a general flowchart of the variance component analysis procedure. The first step is determination of the data attributes including the scaling of the dependent variable(s) and the identification of potential contributing factors or covariates, both as part of the experimental design and as recorded ancillary variables. With regard to the dependent variable, the goal of this step is to understand whether or in what scale linearity is present (or not), and whether the variance is homogeneous across the range of the observations. For the factors and covariates, the goal is to determine whether the model needs to apply exclusively to the specific individuals/settings for each factor (“fixed effects”) or whether the model is intended to generalize beyond these (“random effects”). Discrete covariates are often treated as factors; for continuous covariates the question is whether these are measured with only negligible error, as is taken to be the case here.

The second step is to write down an initial model that includes the putative factors and that represents their interrelationsships. An interrelationship can be the *interaction* between two or more factors (e.g., the differences between results for two sites are not comparable for different peptides - hence site *peptide interaction effect). Or, one factor may be *nested* in another, for example when individual sites have different baselines from which each calculates its own variance. Multi-site experiments typically exhibit both kinds of interrelationships.

Now the model can be fitted (i.e., calculate the estimated parameter/effect values) and tested. Testing has two aspects. Verification that the model fits the data includes identifying outliers, identifying heterogeneity of variance across the range of observations, checking the residuals to look for patterns that should be explained. Then, testing the parameter/effect estimates for statistical significant follows.

The third step is to revise the model to omit factors, interactions and covariates that do not contribute systematically to the value of the dependent variable. Variances need to be recomputed at this point (omitting any extreme outliers); residuals need to be reexamined for definable patterns; and especially if the model is complicated, the significant terms can be investigated further individually using statistical tests. This step is important because, for example, a “significant interaction” between two factors might either occur throughout the experiment or simply occur in just one or two particular cases without being present otherwise (e.g., a difficult-to-measure peptide might be very poorly measured at just one site while throughout the rest of the experiment all the sites are quite consistent).

It should be pointed out that this process may be iterative. Especially removal of experimental outliers often increases the resolution for the model-fitting so that trends or other patterns in the residuals become evident, requiring additional revision to the model itself and certainly to the variance (e.g., denominator of F-test, also the degrees of freedom) used in statistical testing for significance. Finally, the checking process culminates in validation of the assumptions implicit in the analysis computations.

The final step will be model finalization. At this step, the variance components of random effects as well as estimates of fixed effects are computed.

The supplement contains additional tables, graphs of residuals and influence analysis to confirm model adequacy and other information which are too extensive to include in the text or that is confirmatory rather than revelatory.

For this study, the analysis process takes place twice in anticipation of potentially important effects of factors/covariates in the data for the analyte, with the contrary expectation for the spike-in control since the control monitors only the experiment conditions and its conduct.

### 3) The data

**Figure 3:**
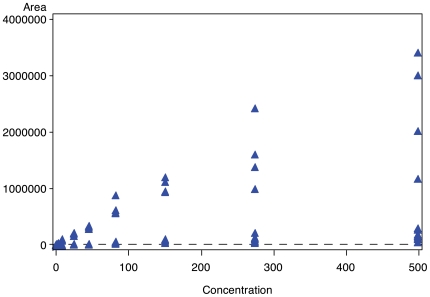
Plot of Area under the peak vs. Concentration for bi0161 (PSA-IVG) acquired in Study I at Site 19, showing the correlation of mean and variance and therefore suggesting log transformation of the data.

Intensity was recorded as (peak) Area for each observation (indexed by sub-study, transition, light or heavy peptide, technical replicate) at each site. The first decision in the variance components analysis is the determination of the appropriate scale of measurement. The plot of Area vs. Concentration in Figure 3 for the light peptide group of one typical peptide bi0161 (PSA-IVG) shows that for Area the variance increases with increasing mean value. Using a log transformation stablizes the variance so that the analyses that follow are not disproportionately driven by values at one end of the range of Areas seen in the study. At the same time, like dilution and many calibration experiments, the concentrations are approximately equally spaced in a log scale. Transforming concentration to the log scale also serves to help equalize the influence of the concentrations at the ends and the middle of the concentration range.

**Figure 4:**
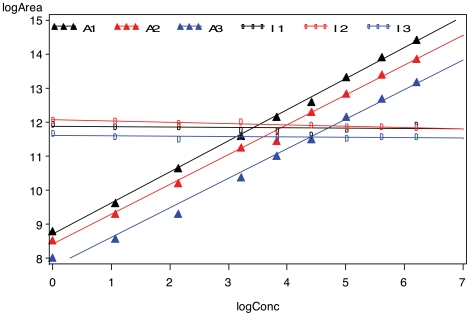
Mean values of logArea vs. logConcentration of PSA-IVG (A, triangles) and its isotope (I, circles) in Study I (1, black), Study II (2, red) and Study III (3, blue). In the log-log scale, the light peptides' relationship to concentration is linear. The heavy peptides (isotopes) served as internal standard with constant concentration as shown.

The effectiveness of the log transformation is illustrated in Figure 4 by the linear relationship of the mean logArea vs. logConcentration for the light peptide (PSA-IVG) and for its isotopic/heavy counterpart. Also, stabilizing the variance and controlling the influence of individual concentrations (especially at the extremes) satisfies the requirements for correct variance component analysis (and other analysis of variance methodology). Therefore all the analyses hereafter will be in the logarithm scale. Note that since the log transformation of blank concentration does not exist, in our analysis, we used only the spiked-in concentration levels, which are greater than zero.

A consequence of the log-log transformation is the linearity of the calibration curves for each of the three sub-studies. From Figure 4 the study-to-study differences show clearly as increments in logArea, with slight differences in slope for the internal controls (heavy peptides) or for the calibration test samples (light peptides).

### 4) Variance Component Analysis

The purpose of a variance components analysis of a calibration experiment is to determine, as precisely as the data allow, the calibration curve, i.e., the function that relates the measurement (logArea) to the stimulus (logConcentration), after eliminating the contributions of other factors and/or covariates to the data values. This is accomplished by decomposing the simplistic variance calculations into parts associated with each of the input factors or covariates and with any important interactions among these. This separation extracts the ascribable variation due to sources other than the relationship of primary interest, leaving only this primary relationship to be estimated together with its remaining variation and hence the goodness of fit for this primary relationship.

For this calibration experiment the relationship of primary interest is logArea as a function of logConcentration; other input factors include the specific sub-study, site or laboratory, peptide group and transition nested within peptide group. Allowing for potential site-to-site differences in moving from one sub-study to the next requires an interaction (study*site); similarly the model allows for potential site-to-site differences in peptide measurement, transition-to-transition variability, etc. And, of course, the model includes the “residual error” that cannot be assigned to any of these sources.

Because the complexity of the MRM study increased from Study I to Study III, the variance was also expected to increase. Each *study (i.e.*, *sub-studies)* is considered to be a fixed effect. Other effects are considered to be random, so that the model is mixed (and all interactions involving one or more random effects are *per force* random).

In order to estimate the variance components, it was assumed that the area measurements after logarithm transform (denoted by logArea) followed a multivariate normal distribution. Of course, to analyze the data for the internal standard, there is no logConcentration term in the model because the spike-in was at constant concentration across all samples. So the mixed effects ANOVA model for the heavy peptides had the following form (**Model 1**):
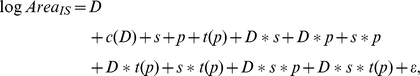
where *study* (*D*) is a fixed effect representing individual sub-studies; *site* (*s*) and *peptide group* (*p*) are random main effects with variances of σ*_s_^2^* and σ*_p_^2^* respectively; *technical_replicate nested within study (c(D))* and *transition nested within peptide group (t(p))* are random nested effects with variance of σ_c(D)_^2^ and σ_t(p)_^2^ respectively; additional random effects include the two-way interactions (study*site <σ*_Ds_^2^*>, study*peptide <σ*_Dp_^2^*>, and site*peptide <σ*_sp_^2^*>), three three-way interactions (study*transition(peptide) <σ*_Dt(p)_^2^*>, site*transition(peptide) <σ*_st(p)_^2^*>, and study*site*peptide<σ*_Dsp_^2^*>*)*, and one four-way interactions (study*site*transition(peptide) <σ*_Dst(p)_^2^*>); *ε* denotes the *residual error* term assuming *ε* to be iid (independent and identical distribution) Gaussian with mean 0 and variance σ_r_^2^.

In this model, with *site* as a random effect, one consequence is that there is a common correlation among all observations within a site. When declaring *peptide group* as a random effect, there is also a common correlation among all observations for the same peptide group. Likewise, the higher order interaction random effects assume common correlations between all observations that had the same level of the corresponding combination of factors. A multivariate model with this covariance structure provides a reasonably good fit to logArea data. The unknown parameters in Model 1 are estimated via restricted/residual maximum likelihood (REML) algorithm [9]. Wald *Z*-tests [10] are used to test the significance of the covariance.

For the light peptides, in addition to the four class-type factors (*study*, *site*, *peptide group* and *transition nested within peptide group*) considered in Model 1, continuous variable of primary interest, i.e., the *concentration* level (log scale, denoted by logConc) is added to the model. As shown in Figure 4, the relationship between the Area and the Concentration is linear in log scale, so for the light peptides (i.e., the analytes, *A*) alone, the full model is (**Model 2**):
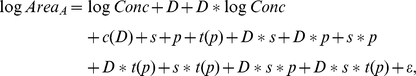
where *logConc*, *study (D)* and the interaction between study and logConc are fixed effects; again, *site (s)* and *peptide group (p)* are random main effects; *technical replicate nested within study (c(D))* and *transition nested within peptide group (t(p))* are random nested effects; and additional random effects are the same as in Model 1; ε, the residual error, is assumed to be iid Gaussian with mean 0 and variance σ_r_^2^. Including the study (D) term in the model allows the intercept of the linear regressions to vary from sub-study to sub-study. By including the interaction term between study and logConc, the slopes of the linear regression also vary with respect to the sub-studies.

In this multi-site study, the measurement of heavy peptides also enabled an alternative analytic approach: to model the area ratio of the light peptides (*A*) to the correponding isotopic counterparts (*IS*). In the log scale, this is equivalent to subtracting the logArea values of the heavy peptides from those of the light peptides. The model takes the following form (**Model 3**): 
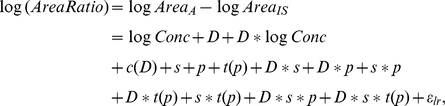
note that *ε_lr_* is the residual error for measurements of the log ratio. Other notations follow those in Model 2.

Models 1, 2 and 3 all assume the homogeneity of variance among peptide groups, which has a common form as follows:
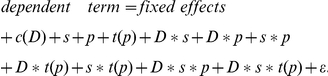



To allow for heterogeneity of variance among peptide groups, the following simplified mixed effects model formula is considered (**Models 4–6**): 
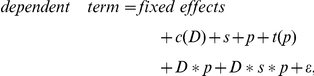
where in the SAS implementation the group effect is specified to be *peptides*. The covariance matrix is thus block diagonal with blocks corresponding to the individual levels of factors containing peptide groups. Other notations are as before.

Similarly, to allow for heterogeneity of variance among sites, the following simplified mixed effects model formula is considered (**Models 7–9**):
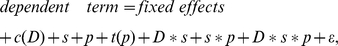
where in the SAS implementation the group effect is specified to be *sites*. The covariance matrix is thus block diagonal with blocks corresponding to the individual levels of factors containing site. Other notations are as before.

The treatments of factors in these models are summarized in the Figure S1.

## Results

**Figure 5:**
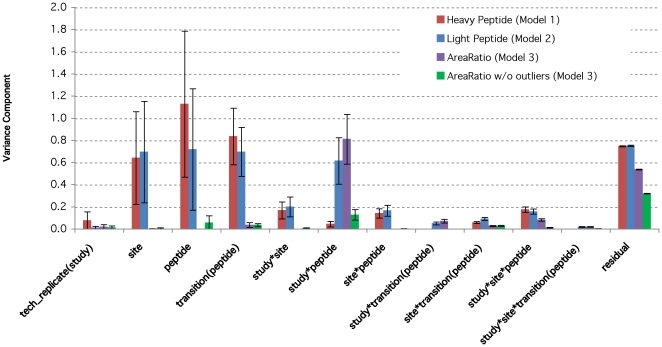
Variance components of Model 1 for heavy peptide data, of Model 2 for light peptide data alone, and of Model 3 for Area Ratio data using whole set and using the subset excluding two peptides ni0001 and bi0170. The error bars represent the standard errors.

The significant level in our study is set at 0.05. Figure 5 summarizes the results (individual variance components and associated standard errors) for Models 1 through 3. Comparison between Model 1 (for heavy peptides) and Model 2 (for light peptides) shows that the variability estimates are generally quite consistent for the random effects. For example, the residual errors for the two analyses were comparable (0.7511 vs. 0.7545). The variance components of *site* are also comparable (0.6446 vs. 0.6983). The variance component of *peptide* is smaller in Model 2. However, for the interaction term of study*peptide, Model 2 has larger variance component (0.82 times of the residual variance, significantly greater than 0 based on Wald test in Table S2) than Model 1 (0.06 times of the residual variance, significantly greater than 0 based on Wald test in Table S1). The variance components of *transition nested with peptide* are significantly greater than 0.

Models 2 and 3 are both for the analysis of the light peptides. They differ in the dependent variables: Model 2 considers the logArea of the light peptides alone, whereas Model 3 considers the log area ratio of light peptides vs. heavy peptides. Comparison of the variance components of these two models (Figure 5) shows that the residual error of Model 3 is smaller than that of Model 2 and the majority of the factors have diminished variance components. In Model 3, the study*peptide term is the dominant variance component (both large and significant), whose variance relative to residual variance is 1.51. It suggests that the peptide groups may play an important role in the variance component analysis of the study. This motivates the new models that allow for different variances for different peptide groups.

**Figure 6:**
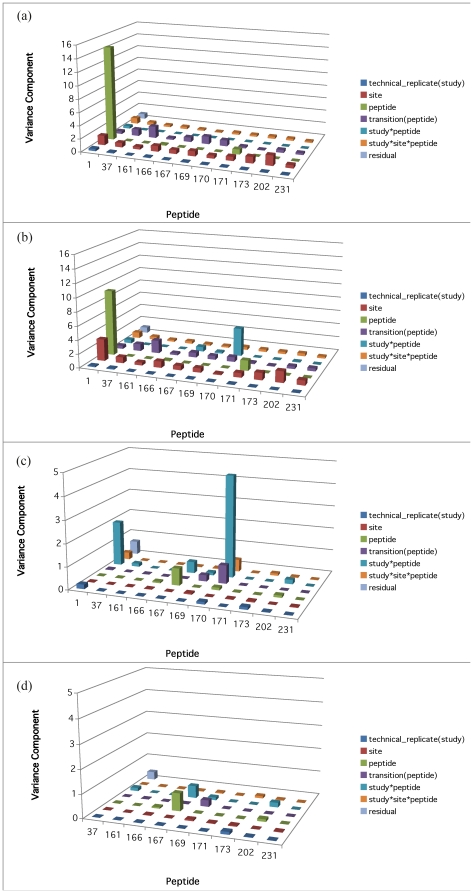
Variance components of models allowing for the heterogeneity of variance among peptide groups. Panels (a)–(c) correspond to Models 4 to 6 respectively, and Panel (d) corresponds to Model 6 without Peptides ni0001 and bi0170.

**Figure 7:**
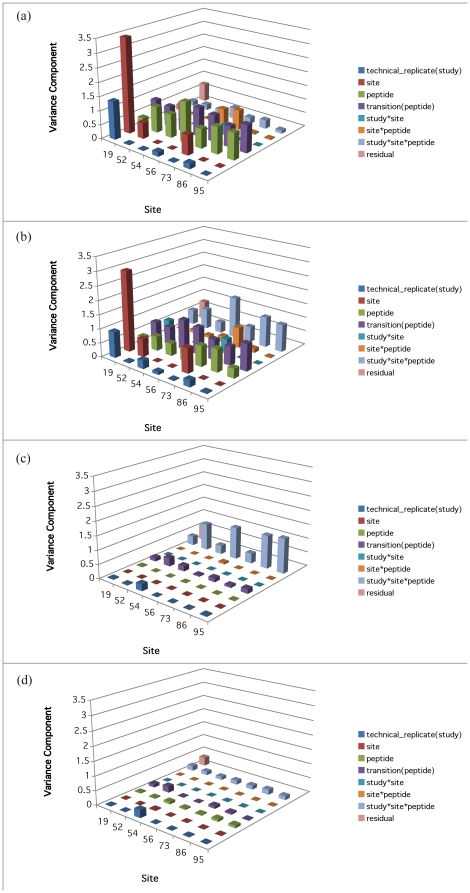
Variance components of models allowing for the heterogeneity of variance among sites. Panels (a)–(c) correspond to Models 7 to 9 respectively, and Panel (d) corresponds to Model 9 without Peptides ni0001 and bi0170.

Models 4 to 6 allow for the heterogeneity of variance among peptide groups for the heavy peptides, light peptides and the area ratio, respectively. Their variance components results are shown in Figure 6 (a) to (c). In Figure 6 (a), there is one predominantly high *peptide* component corresponding to Peptide ni0001 (14.4118, which is 18.55 times of the residual variance). In Figure 6 (b), the same component of Peptide ni0001 remains high (9.4748, which is 11.30 times of the residual variance). In addition, the ‘study*peptide’ component of Peptide bi0170 is also high (4.82 times of the residual variance). In Figure 6 (c) The peptide component corresponding to Peptide ni0001 is diminished, but its ‘study*peptide’ component has increased. Moreover, the ‘study*peptide’ component of Peptide bi0170 remains high. Consistently, on Figure S4 of restricted likelihood distance plot showed that deleting peptide bi0170 resulted in the dramatically increased restricted likelihood distance from others. It seems that Peptides ni0001and bi0170 are the major contributors to the large variability across peptides either directly or through interaction with study or both. Therefore Model 3 and Model 6 are also refitted without these two outlier peptides. The corresponding results are shown in Figure 5 and Figure 6 (d). As expected, excluding the pair of more variable peptides results in the reduction in the variance attributable to the *residuals*and the *site* effect. In addition, three more models (Models 7 to 9) are considered for the heterogeneity of variance among sites. Their variance components results are shown in Figure 7 (a) to (c). The result corresponding to Model 9 without the two outlier peptides are shown in Figure 7 (d).

The ANOVA table of fixed effects of Model 3 excluding Peptides ni0001 and bi0170 is given in Table 1. Both the intercepts and slopes of the linear regressions differ significantly among sub-studies. Further analysis using Student t-test provides for each sub-study the estimates of fixed effects, which are tabulated in Table 2. What's reported in the table are the raw p-values using Study III as reference. The three studies has decreased slope from Study I to Study II to Study III. The intercept of Study I is significantly higher from Study III, whereas the intercept of Study II is not significantly different from Study III.

**Table 1:** F-test of fixed effects in **Model 3** for area ratio excluding Peptides ni0001 and bi0170.

Effect	Numerator Degrees of Freedom	Denominator Degrees of Freedom	F Value	Pr > F
logConc	1	3.30E+04	2.48E+05	<.0001
study	2	8.92	4.62	0.042
logConc*study	2	3.30E+04	2.59E+02	<.0001

**Table 2:** Estimate of fixed effects using **Model 3** for area ratio excluding Peptides ni0001 and bi0170.

Effect	Study	Estimate	Standard Error	t Value	Pr > |t|
Intercept		−4.0570	0.1765	−22.99	<.0001
logConc		0.8426	0.0021	409.47	<.0001
study	I	0.709	0.2334	3.04	0.0122
study	II	0.2999	0.2334	1.29	0.2271
study	III	0	.	.	.
logConc*study	I	0.0862	0.0041	21.29	<.0001
logConc*study	II	0.0538	0.0041	13.23	<.0001
logConc*study	III	0	.	.	.

The covariance parameter estimates and the Wald Z-test of heavy peptides using Models 1–3 are displayed in Tables S1, S2, S3, respectively. The null hypothesis of the Wald Z-test is the hypothesis of null contribution, i.e., the variance component equals to zero. So the null hypothesis is rejected for small p-values. That is, the variance of a specific component was significantly greater than zero. For example, the variances of the residual in Models 1 to 3 are significantly greater than zero. Note that the p-values in our paper were just reported based on the ANOVA analysis, and no model selection or variable selection was attempted based on p-values.

## Discussion

Two objectives motivate this paper: to further illuminate the results of the inter-lab study, and to demonstrate the capabilities and value of mixed effects models for analyzing proteomics data.

Figure S1 provides a context for the discussion by chronicling the information elucidated at each step of the variance components analysis for the dataset in the Addona *et al.* paper.

Mixed effects models are a methodology of choice because they can incorporate complex experimental designs with multiple factors into the analysis; and hence are applicable to multi-factor and multi-site experiments. These models separate the variability due to the different experimental factors and to the residual noise. Moreover, they can model the higher-order interaction terms in a directly interpretable way, which other approaches such as Principle Component Analysis (PCA) cannot. As occurred in the example here, the impact of a factor may appear through an interaction even more prominently than through its direct effect. Through mixed effects modelling, the correlation structure of the data can be explicitly examined by researchers. The flexibility of mixed effects models combined with their inferential power make them a unique and very valuable tool in mass spectrometry-based proteomics research, as is demonstrated through the analysis of this multi-site dataset.

Several characteristics of the experimental design in this study may cause problems or result in faulty interpretation for other analysis tools while variance components analysis via mixed effects models can even take advantage of the design to provide additional information to the researchers. The multilab study was designed with increasing complexicity from Study I to Study III. Therefore in the mixed effects models the *study* factor is treated as a fixed effect. For the heavy peptides, four additional class-type factors (*technical replicates nested within study*, *sites*, *peptides* and *transitions nested within each peptide*) are modeled as random effects, and higher order interaction terms are also included. Modelling these random effects allows for the correlation among all observations that share the same level of each factor. Additionally, by considering a factor (e.g, *sites*) as a random effect, the inference can be applied to an entire population (e.g., experimental sites in general) rather than being restricted to only the particular subsets of the data (e.g., the seven sites). It should be noted here that the purpose of the Addona *et al.* study was to establish broad reproducibility that would be relevant to high quality labs in general. If the goal had been different, for example, to characterize or to calibrate the seven sites, as might be done for an expanded future series of studies, then the sites would have been considered fixed. Of course, the appropriate analysis with sites considered fixed, would give the same variance estimates for the remaining terms. Only site*study would be a fixed interaction effect rather than a random interaction effect (the interaction of site with the other random factors would lead to random interactions).

A second aspect of the study design is that the signals of both the analytes (i.e., light peptides) and their isotopic counterparts (i.e., heavy peptides) are recorded for all experimental units in this study, and the heavy peptides serve as internal standard of the experiment. Thus the variance components analysis for the internal standard can serve to identify sources of variation that are part of the experimental procedures, and simultaneously to provide baseline evidence on the quality of the experiment. Essentially, the variance components analysis for the analytes distinguishes the pervasive sources of experimental variation from variation that is specific to the test material. In the cases of both analyte and heavy isotope, the variance components analysis proceeds through a similar series of steps. Firstly, terms in the model (effects) are identified that contribute the largest components to the variance (or equivalently, extract the most overall variation leaving the least residual variance). Next, if those effects can be subdivided, then the most significant ones (greatest contributors) are identified in order to focus special attention on the possibilities that either the significance of the result depends on a single specific sub-component or that an outlier observation distorts the importance of one term in the model. Discovery of an outlier or of an observation with excessive influence may then lead to deletion of observation(s) and refitting the model, to be followed by verification of the model's goodness of fit to the data via examination of residuals and consideration of influential observations, etc.

The separation of variances arising from different experimental factors and from the residual noise is achieved as follows. From the successfully fitted model, the contributions to variation are quantified in terms of the reduction in total variation (R-squared) that is assignable to each effect; interactions are represented in terms of the covariances between each pair of effects. Then F-tests are used for significance testing; or in the case of single degree-of-freedom contrasts their counterpart t-tests are equivalent.

For the control data based on Model 1, the failure to find significance for *sites* or for *technical replicates* is indicative of a well-controlled experiment. In the case of the multilab study, this is precisely what happens, as is evident in Figure 5. However, the variance components related to the factors of *peptides* and *transition nested within peptide* are much larger than other higher-order interaction terms when compared to the residuals. Moreover, the significance for *transitions nested within peptide* effects is high, which is quite unexpected. Model 4 allows for the variances among different peptides to be different, and its corresponding result in Figure 6 (a) for the control data shows that the high variability of *peptides* was primarily driven by a single very high component corresponding to Peptide ni0001. Model 7 allows for the heterogeneity of variance among different sites. Its result in Figure 7 (a) for the control data shows that Site 19 has higher ‘*site*’ variance component than the other sites.

For the analysis of light peptides alone, the relationship of the peak area values with the concentration levels is linear in the log-log scale. To allow for the differences that could arise from changing SOP, the multiple factor mixed model to fit the compiled data from several sub-studies, allows the intercept and the slope to change from one sub-study to the next. The variance components analysis of this model gives similar results to the one in Model 1 for heavy peptides. For example, even though the ‘*peptide*’ component is smaller for the analysis of light peptides alone, it is offseted by larger ‘study*peptide’ component.So the overal peptide effect remain similar. By looking into the hetergeneous peptide components model of light peptides (Model 5), Peptide ni0001 still gives a much higher ‘*peptide*’ component than others. Moreover, Peptide bi0170 comprises a major ‘study*site’ variance component. From the heterogeneous *site* components model of light peptides (Model 8), it seems that Site 19 still has higher ‘*site*’ variance component than the other sites. And comparing again Figure 7 (a), in Figure 7 (b) the decreased ‘*peptide*’ components are offseted by the increased ‘study*site*peptide’ components.

Utilizing the heavy peptide signals as an internal standard (by calculating the peak area ratio, or in log scale, subtracting the logs of heavy peptide measurements), removes this source of experimental variability. Thus as shown in Figure 5, the remaining variability of the light peptide measurements truly due to the majority of factors is seen to be effectively reduced with the exception of ‘study*peptide’ component. By checking the heterogeneous *peptide* components model of the logAreaRatio (Model 6), the high variability of ‘study*peptide’ is due to Peptides ni0001 and bi0170. The result based on the heterogeneous site components model of the logAreaRatio (Model 9) is shown in Figure 7 (c). Comparing it against Figure 7 (a) and (b), it's impressive to see that the majority of the variance components reduce greatly, including those for Site 19. The only remaining high components are those ‘study*site*peptide’ interactions. This demonstrates the effectiveness of using heavy peptide signals as an internal standard.

In summary, after considering the Area Ratio of light peptides vs. heavy peptides, the variability of measurements related to factors involving *peptides* persist with respect to the residuals. The question then arises whether there is general variability among peptide groups or whether one or a few peptides exhibit dramatically different behavior from the rest of the groups. Based on the evidence so far, the variability corresponding to light peptide groups ni0001 and bi0170 is much higher than the other peptides. They correspond to the peptides of CRP-YEV and MBP-YLA, respectively. Addona *et al.* [6] also found CRP-YEV (ni0001) to be problematic and excluded it from analysis. This concords with the finding here that its variance component is much higher than those of other peptides when considering either light (Figure 6 (a)) or heavy peptides alone (Figure 6 (b)). Using area ratio as the variable of interest actually greatly diminishes the variability for this peptide, as is shown in Figure 6 (c) where the plots are in smaller scale than in Figure 6 (a) and (b). Yet Figure 6 (c) shows that both peptides ni0001 and bi0170 have higher variance components corresponding to the interaction term of study* peptide. Therefore, they are considered peptide outliers.

Following removal of the two peptide outliers, the variance components analysis yields no significant random effects factors with respect to the residuals (Figure 5). The result based on the heterogeneous *site* components model of the logAreaRatio (Model 9) without the outlier peptides is shown in Figure 7 (d). Comparing it to Figure 7 (c), the previously high ‘study*site*peptide’ variance components are reduced. While all other components remain very small, meaning that the two outlier peptides contribute essentially all the excess variation for the *peptide* factor. In addition, the components are quite consistent across different sites except that Site 54 has higher ‘*tech_replicate(study)*’ component than others. The restricted likelihood distance vs. deleted site plot using Model 3 without the two outlier peptides ni0001 and bi0170 is shown in Figure S5, also pointing out that Site 54 does in fact differ from other sites.

The ANOVA table in Table 1 shows that the intercepts and slopes of the calibration curves are different among sub-studies; and Table 2 provides more information about the fixed effect estimates for each sub-study. Slopes of logArea vs. log Concentration plots represent the sensitivity of the proteomics procedure. Table 2 shows that the slopes decrease from Study I to Study II to Study III, as expected, because the complicated digestion process would likely impair the sensitivity of the proteomics procedure.

As is shown above, mixed effects modeling provides powerful inference to this dataset. Yet, care needs to be taken to use it properly, since it has some basic assumptions, which are usually checked in the first step of the variance component analysis (Figure 2). One assumption is that variances are stablized for a range of predictive variables. In the multilab dataset, the raw data of areas under the peaks exhibits correlation between means and variances at different peptide concentrations. Therefore the raw data are transformed into log scale to stablize the variance over a wide range of concentration levels. The residual plots for Model 2 and Model 3 are shown in Figures S2 and S3, respectively.

Model selection is an important aspect of using mixed effects models properly. Depending on the data, either linear or generalized linear models can be used. The multilab dataset, as shown in Figure 4, exhibits a general linearity between logArea and log Concentration. A companion study is underway to study the individual linearity of the calibration curves.

The data considered here came from the seven sites that used the same mass spectrometry platform. Mixed effects modeling methodology can be easily expanded to apply to ensemble data from different platforms. Of course models become more complex with added terms and interactions to investiagate the contributions of various platforms to differences in results. A difficult problem can arise in this comtext when platforms are not replicated at different sites or when the experiment design becomes unbalanced with respect to platform and any other feature (e.g., SOP). In such cases, it can be impossible to distinguish the effect of the individual site from the effect of the platform; it does become impossible when the site used it uniquely.

In conclusion, mixed effects modeling is a valuable tool in mass spectrometry-based proteomics research. The primary purpose of these studies conducted by the multilab team is to test the consistency in the Multiple Reaction Monitoring (MRM) measurements of proteins in human plasma across multiple sites. Results from mixed effects modeling show that the variance attributable to the factors involving *sites* is very small with respect to the residuals when the log area ratio of the light peptides to the heavy peptides is the metric. In other words, overall the MRM measurements are consistent across the labs given that they follow the same standard operating procedures. In addition, the heavy peptide can be used as an effective internal standard for reducing apparent inter-site variability.

## Supporting Information

Table S1 Covariance parameter estimates and Wald Z-test of heavy peptide data using Model 1.
(0.03 MB PDF)

Table S2 Covariance parameter estimates and Wald Z-test of heavy peptide data using Model 2.
(0.03 MB PDF)

Table S3 Covariance parameter estimates and Wald Z-test of heavy peptide data using Model 3.
(0.03 MB PDF)

Figure S1 Flowchart of variance component analysis for the multi-lab dataset.
(1.83 MB TIF)

Figure S2 Residual plots using Model 2 where the peak area of the light peptides is the dependent variable.
(0.08 MB TIF)

Figure S3 Residual plots using Model 3 where the peak area ratio of the light peptides to the heavy peptides is the dependent variable.
(0.07 MB TIF)

Figure S4 Restricted likelihood distance vs. deleted peptide plot using Model 3 where the peak area ratio of the light peptides to the heavy peptides is the dependent variable.
(0.04 MB TIF)

Figure S5 Restricted likelihood distance vs. deleted site plot using Model 3 without the two outlier peptides ni0001 and bi0170.
(0.04 MB TIF)

## References

[1] Issaq HJ, Veenstra TD, Conrads TP, Felschow D (2002). The SELDI-TOF MS approach to proteomics: protein profiling and biomarker identification.. Biochemical and Biophysical Research Communications.

[2] Diamandis EP (2004). Mass spectrometry as a diagnostic and a cancer biomarker discovery tool.. Molecular & Cellular Proteomics.

[3] Wang J, Wei Y, Wang D, Chan LL, Dai J (2008). Proteomic study of the effects of complex environmental stresses in the livers of goldfish (Carassius auratus) that inhabit Gaobeidian Lake in Beijing, China.. Ecotoxicology.

[4] Bell AW, Deutsch EW, Au CE, Kearney RE, Beavis R (2009). A HUPO test sample study reveals common problems in mass spectrometry-based proteomics.. Nature Methods.

[5] Eckel-Passow JE, Oberg AL, Therneau TM (2009). An insight into high-resolution mass-spectrometry data.. Biostatistics.

[6] Addona TA, Abbatiello SE, Schilling B, Skates SJ, Mani DR (2009). Multi-site assessment of the precision and reproducibility of multiple reaction monitoring-based measurements of proteins in plasma.. Nature Biotechnology.

[7] Mercier C, Truntzer C, Pecqueur D, Gimeno JP, Belz G (2009). Mixed-model of ANOVA for measurement reproducibility in proteomics.. Journal of Proteomics.

[8] Box GE, Hunter JS, Hunter WG (2005). Statistics for experimenters: design, innovation, and discovery.. Wiley.

[9] Harville DA (1977). Maximum likelihood approaches to variance component estimation and to related problems.. Journal of the American Statistical Association.

[10] Sprinthall RC (2003). Basic statistical analysis: seventh edition.. Pearson Education Group.

