# Supplementary material for: Variance Component Analysis of a Multi-Site Study for the Reproducibility of Multiple Reaction Monitoring Measurements of Peptides in Human Plasma
Source: PLoS One. 2011 Jan 26;6(1):e14590. doi: 10.1371/journal.pone.0014590 (PMC3027641; doi:10.1371/journal.pone.0014590)
Supplement: Table S1 — Covariance parameter estimates and Wald Z-test of heavy peptide data using Model 1. (0.03 MB PDF) [file pone.0014590.s001.pdf]

Table S1. Covariance parameter estimates and Wald Z-test of heavy peptide data using **Model 1**.

**Covariance Parameter**	**Estimate**	**Standard Error**	**Z Value**	**Probability > Z**
tech_replicate(study)	0.0795	0.0795	1	0.1589
site	0.6446	0.4183	1.54	0.0617
peptide	**1.1307**	**0.6591**	**1.72**	**0.0431**
transition(peptide)	**0.8387**	**0.2559**	**3.28**	**0.0005**
study*site	**0.1716**	**0.0771**	**2.23**	**0.013**
study*peptide	**0.0477**	**0.0248**	**1.92**	**0.0273**
site*peptide	**0.1445**	**0.0423**	**3.42**	**0.0003**
study*transition(peptide)	**0.0011**	**0.0007**	**1.73**	**0.0419**
site*transition(peptide)	**0.0618**	**0.0081**	**7.66**	**<.0001**
study*site*peptide	**0.1790**	**0.0243**	**7.37**	**<.0001**
study*site*transition(peptide)	**0**	**.**	**.**	**.**
residual	**0.7511**	**0.0046**	**162.28**	**<.0001**
