# Supplementary material for: Variance Component Analysis of a Multi-Site Study for the Reproducibility of Multiple Reaction Monitoring Measurements of Peptides in Human Plasma
Source: PLoS One. 2011 Jan 26;6(1):e14590. doi: 10.1371/journal.pone.0014590 (PMC3027641; doi:10.1371/journal.pone.0014590)
Supplement: Table S2 — Covariance parameter estimates and Wald Z-test of heavy peptide data using Model 2. (0.03 MB PDF) [file pone.0014590.s002.pdf]

Table S2. Covariance parameter estimates and Wald Z-test of light peptide data using **Model 2**.

**Covariance Parameter**	**Estimate**	**Standard Error**	**Z Value**	**Probability > Z**
tech_replicate(study)	0.0144	0.0145	0.99	0.1603
site	0.6983	0.4571	1.53	0.0633
peptide	0.7213	0.5477	1.32	0.0939
transition(peptide)	**0.6995**	**0.2210**	**3.16**	**0.0008**
study*site	**0.2027**	**0.0893**	**2.27**	**0.0116**
study*peptide	**0.6181**	**0.2092**	**2.95**	**0.0016**
site*peptide	**0.1693**	**0.0493**	**3.44**	**0.0003**
study*transition(peptide)	**0.0537**	**0.0126**	**4.27**	**<.0001**
site*transition(peptide)	**0.0948**	**0.0135**	**7.04**	**<.0001**
study*site*peptide	**0.1605**	**0.0232**	**6.92**	**<.0001**
study*site*transition(peptide)	**0.0223**	**0.0034**	**6.65**	**<.0001**
residual	**0.7545**	**0.0054**	**139.22**	**<.0001**
