# Supplementary material for: Variance Component Analysis of a Multi-Site Study for the Reproducibility of Multiple Reaction Monitoring Measurements of Peptides in Human Plasma
Source: PLoS One. 2011 Jan 26;6(1):e14590. doi: 10.1371/journal.pone.0014590 (PMC3027641; doi:10.1371/journal.pone.0014590)
Supplement: Table S3 — Covariance parameter estimates and Wald Z-test of heavy peptide data using Model 3. (0.03 MB PDF) [file pone.0014590.s003.pdf]

Table S3. Covariance parameter estimates and Wald Z-test of Area Ratio data using **Model 3**.

**Covariance Parameter**	**Estimate**	**Standard Error**	**Z Value**	**Probability > Z**
tech_replicate(study)	0.02155	0.02162	1	0.1594
site	0.00374	0.0045	0.84	0.2018
peptide	0	.	.	.
transition(peptide)	**0.0400**	**0.0221**	**1.81**	**0.0354**
*study*site*	0	.	.	.
study*peptide	**0.8138**	**0.2243**	**3.63**	**0.0001**
*site*peptide*	0	.	.	.
study*transition(peptide)	**0.0746**	**0.0170**	**4.4**	**<.0001**
site*transition(peptide)	**0.0308**	**0.0053**	**5.82**	**<.0001**
study*site*peptide	**0.0861**	**0.0111**	**7.79**	**<.0001**
study*site*transition(peptide)	**0.0232**	**0.0030**	**7.64**	**<.0001**
residual	**0.5403**	**0.0039**	**138.91**	**<.0001**
