# Supplementary figures and images for: Variance Component Analysis of a Multi-Site Study for the Reproducibility of Multiple Reaction Monitoring Measurements of Peptides in Human Plasma
Source: PLoS One. 2011 Jan 26;6(1):e14590. doi: 10.1371/journal.pone.0014590 (PMC3027641; doi:10.1371/journal.pone.0014590)

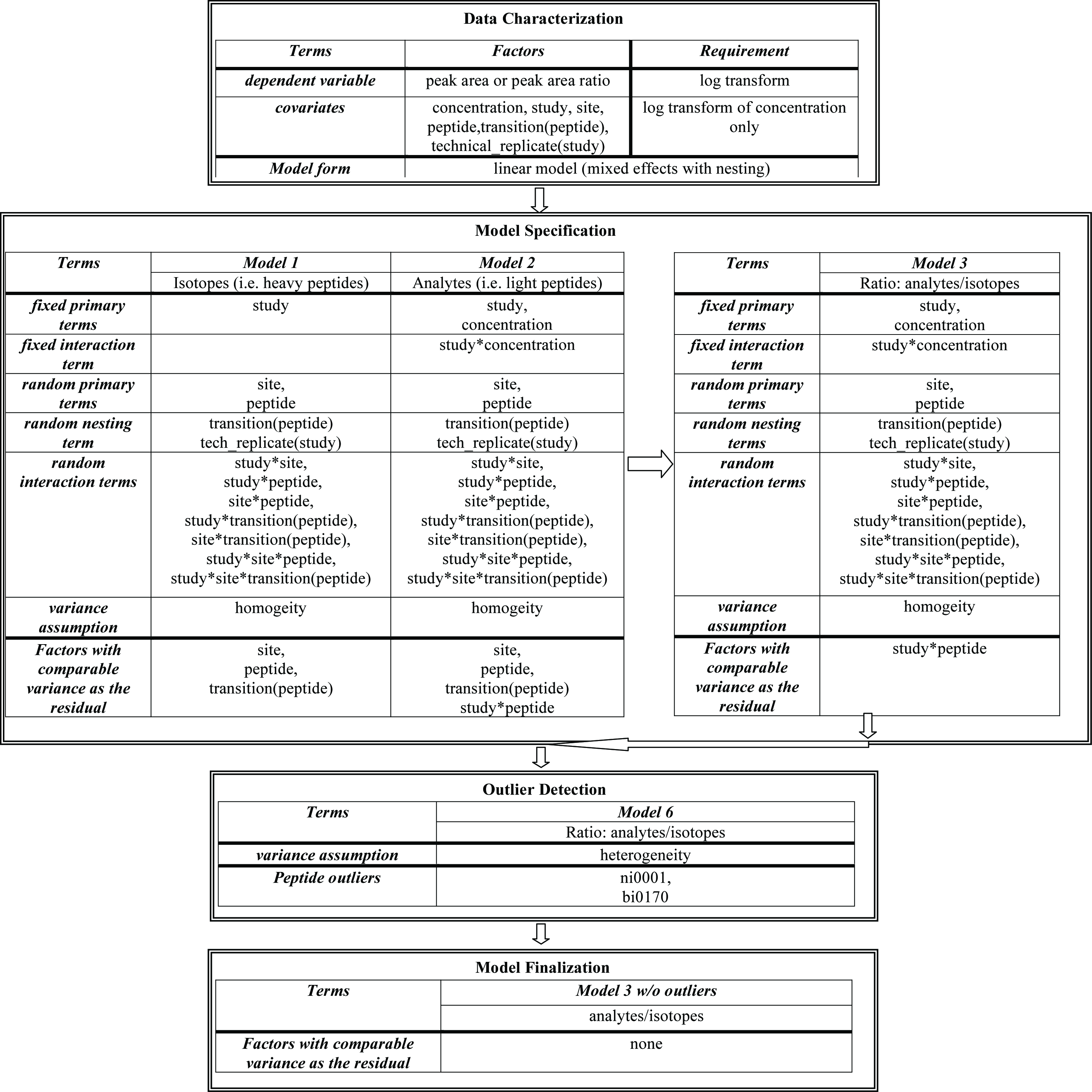

Supplement: Figure S1 — Flowchart of variance component analysis for the multi-lab dataset. (1.83 MB TIF) [file pone.0014590.s004.tif]

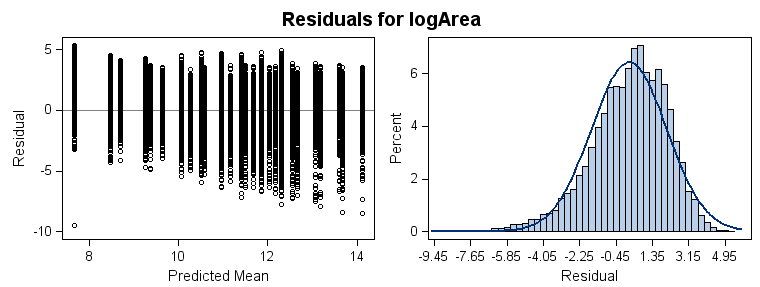

Supplement: Figure S2 — Residual plots using Model 2 where the peak area of the light peptides is the dependent variable. (0.08 MB TIF) [file pone.0014590.s005.tif]

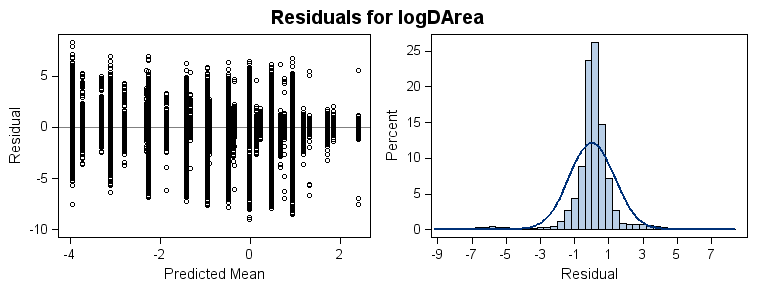

Supplement: Figure S3 — Residual plots using Model 3 where the peak area ratio of the light peptides to the heavy peptides is the dependent variable. (0.07 MB TIF) [file pone.0014590.s006.tif]

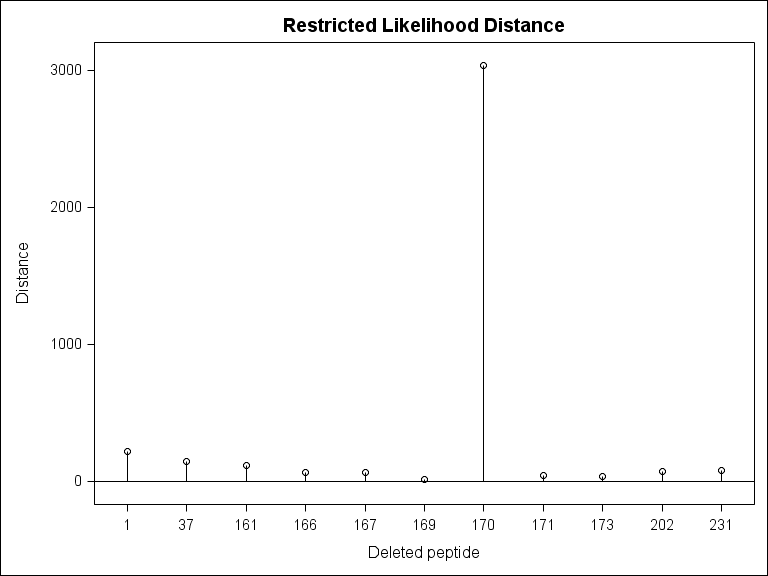

Supplement: Figure S4 — Restricted likelihood distance vs. deleted peptide plot using Model 3 where the peak area ratio of the light peptides to the heavy peptides is the dependent variable. (0.04 MB TIF) [file pone.0014590.s007.tif]

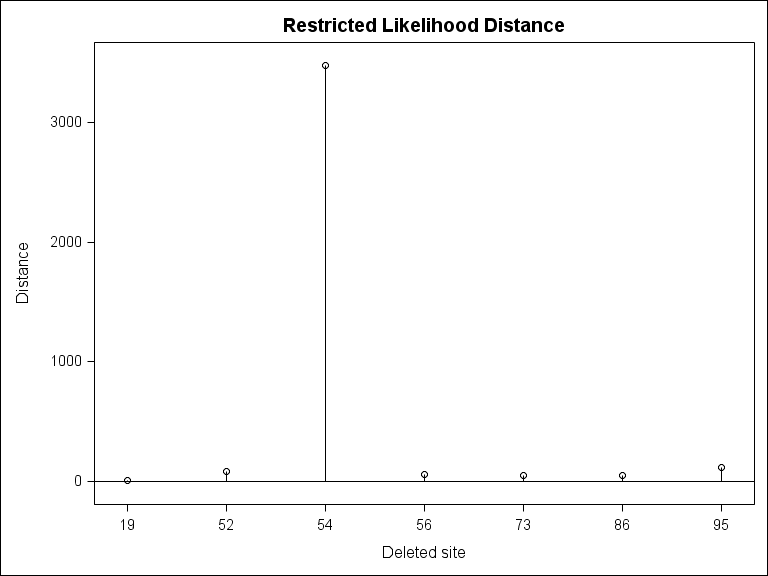

Supplement: Figure S5 — Restricted likelihood distance vs. deleted site plot using Model 3 without the two outlier peptides ni0001 and bi0170. (0.04 MB TIF) [file pone.0014590.s008.tif]
